# Why Do You Drink Caffeine? The Development of the Motives for Caffeine Consumption Questionnaire (MCCQ) and Its Relationship with Gender, Age and the Types of Caffeinated Beverages

**DOI:** 10.1007/s11469-017-9822-3

**Published:** 2017-10-31

**Authors:** Csilla Ágoston, Róbert Urbán, Orsolya Király, Mark D. Griffiths, Peter J. Rogers, Zsolt Demetrovics

**Affiliations:** 1 Institute of Psychology, ELTE Eötvös Loránd University, Izabella utca 46, Budapest, 1064 Hungary; 2 Doctoral School of Psychology, ELTE Eötvös Loránd University, Budapest, Hungary; 3 International Gaming Research Unit, Psychology Department, Nottingham Trent University, Nottingham, NG1 4BU UK; 4 School of Experimental Psychology, University of Bristol, Bristol, UK

**Keywords:** Caffeine consumption, Caffeine drinking motives, Motives for Caffeine Consumption Questionnaire, Energy drink consumption, Psychometric testing

## Abstract

Caffeine is the most popular psychoactive substance that is consumed worldwide. As motives influence behavior, investigation of the motivational background of caffeine consumption should help provide a better understanding of the popularity of caffeinated products. The present study aimed (i) to explore and operationalize the motives of caffeine consumption and (ii) to reveal possible differences in the motives regarding gender, age and the type of caffeinated products consumed. Motives for caffeine consumption were collected from regular caffeine consumers (*N* = 26) and were informed by a review of the relevant literature. Following this, a cross-sectional study was conducted on a convenience sample of Hungarian university students and working adults (*N* = 598). The participants completed the Motives for Caffeine Consumption Questionnaire and the Caffeine Consumption Questionnaire. Six motivational factors were identified: Alertness, Habit, Mood, Social, Taste and Symptom Management. Women had higher scores on Habit, Social, Taste and Symptom Management. Younger participants had higher scores on Alertness than the older group, and the older group had higher scores on Habit and Symptom Management. Five types of caffeine users were identified. Those who consumed (i) coffee, (ii) tea, (iii) energy drinks, (iv) coffee and tea and (v) mixed drinks. Several differences between the five groups were revealed across all motives except for Taste. The present study developed a robust psychometric instrument for assessing caffeine consumption motives. The factors varied in importance in relation to gender, age and caffeine consumption habits.

Caffeine is the most popular psychoactive substance ingested in the world (International Research Agency For Research On Cancer 1991). In the USA, approximately 89% of the adults consume caffeinated beverages on a daily basis (Fulgoni et al. 2015). Caffeine consumption in Hungary is even more prevalent with 91.5% of males (121.7 mg/day) and 93.2% of females (123.1 mg/day) consuming it daily (Szeitz-Szabó et al. 2011). Despite its popularity, relatively few studies deal with the characteristics of caffeine use, and particularly with the underlying motives for consumption. Examining the short- and long-term effects of positive and negative consequences of caffeine use for physical and mental health is also important. For example, caffeine has a known stimulant effect (Barry et al. 2008), and its consumption results in enhanced alertness with faster reaction time (Souissi et al. 2013). Caffeine concentration in the body peaks at between 45 and 60 minutes after ingestion (Krieger et al. 2016).

Several studies have demonstrated that coffee has neutral or slightly beneficial effects on health. For instance, coffee consumption is inversely associated with mortality (Crippa et al. 2014; Je and Giovannucci 2014) and it can decrease cognitive decline (Santos et al. 2010). In contrast, regular caffeine consumption is also associated with a higher risk of hypertension (James 2004; Noordzij et al. 2005). Caffeine has the potential to contribute to the emergence of many psychiatric symptoms, such as symptoms of anxiety or depression (Broderick and Benjamin 2004) and psychosis (Crowe et al. 2011; Jones and Fernyhough 2009; Lucas et al. 1990). Caffeine also has a low (but documented) addictive potential (Heishman and Henningfield 1992). Due to the existing evidence, ‘caffeine withdrawal’ is now included in DSM-5 (American Psychiatric Association 2013). Although caffeine dependence or—in more recent terms—‘caffeine use disorder’ is not included yet as a clinical diagnosis in the DSM-5, it is indicated as a condition for further study (American Psychiatric Association 2013; Meredith et al. 2013).

As described above, there are several physical and psychological advantages and disadvantages of caffeine consumption. Therefore, it is especially important to know the underlying motivations of caffeine use, because motives can be determining factors of behavior (McClelland 1985). The role and the importance of such motives have already been investigated in the case of other substances such as alcohol (Kuntsche et al. 2007) marijuana (Simons et al. 2000), and nicotine (Vajer et al. 2011).

There are various explanations for caffeine consumption. By reputation, caffeine has many appealing characteristics, including attention improvement especially when alertness is low (Brice and Smith 2001). However, with repeated use, tolerance appears to develop to the wakefulness and alerting effects of caffeine, so consumption may also become important for the consumer as a way of alleviating withdrawal symptoms (Griffiths et al. 1986; James and Rogers 2005; Rogers et al. 2013). In other words, both positive and negative reinforcement need to be taken into consideration when examining the motives for caffeine consumption (Heinz et al. 2009).

If individuals are asked why they consume beverages containing caffeine, they often mention the taste (Rogers and Smith 2011). The preference for the bitter (and arguably aversive) taste of caffeine might be associated with the positive effects caused by the psychoactive substance (Rogers and Smith 2011). The sensory qualities of caffeinated beverages, such as taste and smell, may become associated with caffeine-induced mood changes (Rogers and Richardson 1993; Rogers et al. 1995).

In recent studies, the expectations regarding caffeine consumption have been thoroughly examined (Heinz et al. 2009; Huntley and Juliano 2012; Schott et al. 2016). Heinz et al. (2009) documented four different expectancies comprising ‘withdrawal symptoms’, ‘positive effects’, ‘acute negative effects’ and ‘mood effects’. Huntley and Juliano (2012) reported a more detailed, but to some extent similar, set of expectancies: ‘withdrawal/dependence’, ‘energy/work enhancement’, ‘appetite suppression’, ‘social/mood enhancement’, ‘physical performance enhancement’, ‘anxiety/negative physical effects’ and ‘sleep disturbance’. Schott et al. (2016) reported the same seven expectancy factors among German-speaking participants and found that these factors correlated with caffeine use disorder symptoms to varying degrees. Though motives are considered to be more proximal predictors of substance use than expectancies (Kuntsche et al. 2007), the recognition of the caffeine expectancies can lead to a better understanding of the motives of caffeine use. Furthermore, early research by Graham (1988) applied four alcohol consumption motives to caffeine: two personal factors (i.e., ‘stimulant’ and ‘relief’) and two social factors (i.e., ‘sociability’ and ‘beverage’). However, Graham’s study was only based on a deductive method and was conducted before the expansion of energy drinks. Therefore, there could be other motives underlying the consumption of caffeine.

Previous research shows that a distinction can be made across drugs (e.g., alcohol, nicotine, cannabis) and age groups, between the two pathways of anxiety sensitivity/trait anxiety to coping and conformity motives versus intensity seeking to enhancement motives (Comeau et al. 2001). Schepis (2014) suggested that the differences in the correlates of nonmedical use of prescription Zolpidem (NUPZ) in different age groups can be due to the motives underlying NUPZ. It has also been shown that differences in motives of playing online games also emerge between different age groups (Demetrovics et al. 2011). In addition to gender and age, the different types of the caffeinated products also have to be considered. Some studies have focused on one type of caffeinated product such as energy drinks (e.g., Malinauskas et al. 2007) or coffee (e.g., Butt and Sultan 2011) while others have examined all types (e.g., Huntley and Juliano 2012).

The main aim of the present study was to develop a psychometrically robust measure for assessing caffeine consumption motives (i.e., the Motives for Caffeine Consumption Questionnaire). Both inductive and deductive methods were applied in its construction. The secondary aim of the study was to examine the possible emerging motivational differences regarding gender, age, and the type of the caffeinated beverages consumed as an initial step in the validation of the measure.

## Method

### Pilot Study

A pilot study formed the inductive phase of the construction of the Motives for Caffeine Consumption Questionnaire (MCCQ). In this pilot study, 26 regular caffeine consumers (13 males and 13 females) were recruited by convenience sampling and interviewed about their motives of caffeine consumption. The mean age of the participants was 30.69 years (SD = 11.69) and the mean of the daily caffeine consumption was 262.88 mg (SD = 217.82). There were no significant differences in daily caffeine consumption between male (*M* = 257.29 mg, SD = 239.99) and female (*M* = 280.58 mg, SD = 208.49) participants. On a daily basis, 84.6% of the participants consumed coffee, 57.5% tea, and 23.08% energy drinks. Overall 98 statements were collected this way. Further details of the scale’s construction are described below*.*

### Sample and Procedure

A total of 598 participants completed a questionnaire concerning their caffeine consumption in 2012. The present research involved two user groups: university students (*n* = 400) and working adults (*n* = 198). Only those who consumed caffeinated products in the past month were included in the study. Participants were approached by a research assistant and asked to participate in the study. The questionnaires were administered in paper-based form. For the first group, 400 questionnaires were administered in the building of the Institute of Psychology, Eötvös Loránd University (ELTE), and in a student residence of the ELTE (Budapest, Hungary). Every student who was asked to participate and was a caffeine consumer completed the questionnaire. For working adults, all employees of a company in Western Hungary (specializing in producing complex connection units such as connectors, electromechanical components and cable assemblies) were recruited for participation. During working hours, 250 employees in three facilities of the company were administered the questionnaires. Around one-fifth of those asked (*n* = 52) declined to participate or did not return the questionnaire. No sociodemographic data were collected for those who refused to participate in the study. Participants within the university and the company were chosen using convenience sampling. The data from the two samples were merged for the current study. Participants engaged in the study without any financial or other compensation.

### Measures

#### Demographic Variables

In addition to standard demographic characteristics (gender, age, place of residence, marital status, educational attainment, school, work, subjective socio-economic status), data were collected on the weight and height of the person in order to compute caffeine consumption in proportion to body mass index.

#### Amount of Caffeine Consumed

The extent of caffeine consumption was assessed using the Caffeine Consumption Questionnaire (CCQ) (Landrum 1992). The original table was modified to align the caffeinated products available in Hungary. For this reason, several caffeinated soft drinks and medications that are not available in Hungary were excluded, leaving coffee, instant coffee, tea, energy drinks, caffeine pills, and cola drinks. Because of their low caffeine content, decaffeinated coffee, hot chocolate, and chocolate were not assessed. Participants had to indicate how many portions of each caffeinated product they consumed during a typical day and what time of the day they usually consumed it.

The caffeine content of caffeinated products was calculated on the basis of previous studies (Barone and Roberts 1996; Chin et al. 2008; International Food Information Council Foundation 2008; Mineharu et al. 2009; Roehrs and Roth 2008; Rogers and Smith 2011)—taking the mean of the different calculations—and the packaging information available on energy drinks and caffeine pills. The estimated caffeine content was 100 mg per cup (150 ml) of ground coffee (including espresso, brewed, and drip coffee), 60 mg per cup (150 ml) of instant coffee (powder or granulated form), 50 mg per cup (200 ml) of black tea, 40 mg per cup (200 ml) of green tea, and 30 mg per can (330 ml) of cola. Caffeine pills available on the Hungarian market usually contain 100 mg caffeine per pill, and energy drinks contain 75 mg (based on the caffeine content of 30 selected energy drinks which were chosen for the current calculations).

#### The Development of the Items of Motives for Caffeine Consumption Questionnaire

For the assessment of caffeine consumption motives, participants completed the item pool of the Motives for Caffeine Consumption Questionnaire (MCCQ) that was specially developed for this study. The items of the questionnaire were created through an inductive method that was undertaken during a pilot study (see above for details). The items were then verified and classified by a deductive method, and which included the review of the relevant literature. On the basis of theoretical consideration—mainly based on the findings of Graham (1988)—the responses were categorized into the following topics: habit, ceasing fatigue, invigoration, improving concentration (these three being similar to Graham’s ‘stimulant’ factor), consumption because of the taste or the smell of the beverage (Graham’s ‘beverage’ factor), symptom management, mood (Graham’s ‘relief’ factor) and social reasons (Graham’s ‘sociability’ factor). In some cases, classification was not possible. In these cases, an ‘other’ category was also configured. After the exclusion of external motives related to specific situations, metaphoric phrasings and duplicates, the final list contained 39 items. The study was conducted with the permission of the Research Ethics Committee of ELTE Faculty of Education and Psychology.

### Statistical Analysis

To investigate the factor structure of the MCCQ, a complex examination method was chosen, based on a procedure described by Brown (2006) and used by Brown et al. (2005) and Demetrovics et al. (2011). Because of the smaller sample size, a less complex analysis was applied that comprised an exploratory factor analysis (EFA) and a confirmatory factor analysis (CFA). Regarding the first aim of the study, both the EFA and the CFA were performed with MPLUS 6.0 (Muthén and Muthén 1998–2011). For this procedure, two non-overlapping groups were selected from our sample for the EFA and the CFA. Participants from the merged sample of students and working adults were randomly assigned into two samples (i.e., sample 1 and sample 2) using SPSS. EFA was performed on sample 1 (*n* = 290) to establish the factor structure of the 39-item questionnaire. From sample 1, a modified pool of items (e.g., solutions that excluded problematic items [see ‘Factor structure of MCCQ’ section]) was extracted that provided the factor structure for the CFA that was tested on sample 2 (*n* = 308).

Ratification of the factor solution was based on multiple fit indices, because they provide different information for evaluating model fit. Chi-square as a basic fit index was introduced but due to its sensitivity to larger sample size; other fit indices were relied upon. For both EFA and CFA models, the goodness of fit was evaluated by the Tucker–Lewis Index (TLI) (Tucker and Lewis 1973) and the Comparative Fit Index (CFI) (Bentler 1990). For model evaluation, the study also used the root mean square error of approximation (RMSEA) (Steiger 1990), its 90% confidence interval (MacCallum et al. 1996), and the standardized root mean square residual (SRMR) (Hu and Bentler 1999). Model fit was considered acceptable under the following conditions: CFI (> .90), TLI (> .90), SRMR (< .08), RMSEA < .07 [CI] < .08. An excellent fit is expressed by the following: CFI (> .95), TLI (> .95), SRMR (< .05) and RMSEA (< .05) [CI] < 0.08 (Hooper et al. 2008; Hu and Bentler 1999). Descriptive statistics, *t* tests for the comparison of gender and the two age groups, and ANOVA for the comparison of the types of caffeinated beverages, were performed with SPSS (version 20) (IBM Corp. 2011).

## Results

### Descriptive Statistics

Descriptive statistics of the demographic characteristics and the amount of consumed caffeine for sample 1 (*n* = 290), sample 2 (*n* = 308), and the total sample (*N* = 598) are presented in Table 1. Chi-square tests revealed no significant differences between the two samples regarding gender, place of residence, educational attainment, current studies, and work status. *T* tests revealed significant differences regarding age [*t*(585.341 = 2.36, *p* = 0.019] and average daily caffeine consumption [*t*(591 = 2.21, *p* = 0.027] indicating that participants in sample 1 were slightly younger and had a lower daily caffeine consumption. There were no significant differences in the subjective socioeconomic status between the two groups.

**Table 1:** Demographic characteristics of sample 1, sample 2 and the total sample

Characteristics		Sample 1	Sample 2	Total sample
		(*n* = 290)	(*n* = 308)	(*N* = 598)
Gender *N* (%)	Female	213 (73.4)	214(69.5)	427(71.4)
Age (years) [mean (*SD*)]		26.78 (9.9)	28.83 (11.1)	27.84 (10.6)
Place of residence *N* (%)	Budapest	73 (25.2)	82 (26.6)	155 (25.9)
	Another city	140 (48.3)	160 (51.9)	300 (50.2)
	Village	77 (26.6)	65 (21.1)	142 (23.7)
	Missing	0 (0)	1 (.3)	1 (.2)
Educational attainment *N* (%)	Elementary school	13 (4.5)	9 (2.9)	22 (3.7)
	Vocational school	34 (11.7)	36 (11.7)	70 (11.7)
	High school	196 (67.6)	193 (62.7)	388 (64.9)
	College. university	46 (15.9)	69 (22.4)	115 (19.2)
	Missing	1 (.3)	1 (.3)	3 (.5)
Current studies N (%)	No studies	89 (30.7)	113 (36.7)	202 (33.8)
	Full-time education	195 (67.2)	187 (60.7)	382 (63.9)
	Evening classes	2 (.7)	2 (.6)	4 (.7)
	Another courses	2 (.7)	6 (1.9)	8 (1.3)
	Missing	2 (.7)	0 (0)	2 (.3)
Work status *N* (%)	Unemployed	154 (53.1)	140 (45.5)	294 (49.2)
	Full-time job	92 (31.7)	118 (38.3)	210 (35.1)
	Part-time job	16 (5.5)	15 (4.9)	31 (5.2)
	Less than part-time job	26 (9)	35 (11.4)	61 (10.2)
	Missing	2 (.7)	0 (0)	3 (.2)
Subjective SES [mean (SD)]		3.78 (.75)	3.76 (.72)	3.77 (.74)
Caffeine (mg) per day [mean (*SD*)]		197.1 (140.1)	224.2 (158)	211.1 (150.1)

*SD* standard deviation, SES socioeconomic status

Participants consumed different kinds of caffeinated products and to different extents. The grouping of participants by source of caffeine intake was based on previous studies (e.g., Huntley and Juliano 2012; Schott et al. 2016). When the groups were created, two main aspects were considered. More specifically, they were to (i) handle participants with mixed caffeine consumption habits and (ii) form groups with appropriate number of participants. Finally, five groups of caffeine consumers were created: (1) ‘coffee’ (at least two-thirds of the daily consumption derived from coffee), (2) ‘tea’ (at least two-thirds of the daily consumption derived from tea), (3) ‘energy drink’ (at least two-thirds of the daily consumption derived from energy drinks), (4) ‘coffee and tea’ (the daily consumption comprising coffee and tea where neither of them obtain the two-thirds of the daily consumption) and (5) ‘mixed’ (two or more types of caffeinated products were consumed—except for the combination of coffee and tea—or caffeinated products other than coffee, tea or energy drinks were consumed). Those who consumed other caffeinated products, namely cola (*n* = 9) and caffeine pills (*n* = 1) as the main source of caffeine, were excluded from this analysis due to the very low number of cases. One-third of the participants (36%; *n* = 215) comprised the ‘coffee’ group, 25.4% (*n* = 152) comprised the ‘tea’ group, 3.2% (*n* = 19) comprised the ‘energy drink’ group, 14.5% (*n* = 87) comprised the ‘coffee and tea’ group and 11.7% (*n* = 70) comprised the ‘mixed’ group. The remainder of the respondents (9.2%; *n* = 55) did not answer the question.

### Factor Structure of the MCCQ

During the examination of the factor structure of the MCCQ, specific attention was paid to two criteria. First, acceptability of the factor solution was based on the adequacy of the aforementioned fit indices, second, the interpretability of the solution. As a first step, an exploratory factor analysis was performed with robust maximum-likelihood estimation and geomin rotation to establish the factor structure of the 39 items on sample 1 (*n* = 290). Initially, seven factors with eigenvalues greater than 1 emerged.

The 7-factor solution could not be interpreted, because on factor 2, there were no items with factor loadings greater than 0.60. The 8-factor solution was also rejected because on factor 2 and factor 8, there were no items with factor loadings greater than 0.60. The 6-factor solution provided interpretable factors and had the best goodness-of-fit indices (*χ*2 = 1102.7, df = 522, *p* < 0.001; CFI = 0.913; TLI = 0.877; SRMR = 0.031; RMSEA = 0.062 [CI: 0.057–0.067]) compared to the 4-factor solution (*χ*2 = 1524.1, df = 591, *p* < 0.001; CFI = 0.861; TLI = 0.826; SRMR = 0.041; RMSEA = 0.074 [CI: 0.069–0.078]) and the 5-factor solution (*χ*2 = 1261.2, df = 556, *p* < 0.001; CFI = 0.895; TLI = 0.860; SRMR = 0.036; RMSEA = 0.066 [CI: 0.061–0.071]).

Following these analyses, the 6-factor solution was thoroughly examined. Items were kept based on two criteria suggested by Costello and Osborne (2005) (i) they should have salient factor loadings (> 0.40) and (ii) small cross-loadings (an item loads at less than .32 on other factors). The second criterion was amended with the following condition: difference between the best and second-best loadings should be greater than at least 0.30 in order to keep the item. Seven items were eliminated because they did not meet the first criterion. Because of cross-loading (second criterion), another nine items were dropped. Table 2 presents the factor loadings for the 6-factor solution.

**Table 2:** Exploratory factor analysis of the MCCQ in sample 1 (*n* = 290)

Item	Factor 1	Factor 2	Factor 3	Factor 4	Factor 5	Factor 6
M1 …because it became a ritual for me	**0.806**	− 0.042	− 0.049	0.112	0.006	0.121
M8 …because it became an enjoyable habit	**0.585**	− 0.011	0.144	0.208	0.206	− 0.053
M15 …because it became the part of my everyday life	**0.640**	0.004	0.048	0.013	0.070	*0.357*
M24 …because it is a pleasant ritual	**0.595**	0.040	0.018	*0.306*	0.131	0.003
M2 …because it helps when I am tired	0.089	**0.858**	− 0.074	0.013	− 0.027	− 0.053
M4 …because it helps me to concentrate	0.035	**0.837**	0.081	0.005	− 0.011	− 0.155
M9 …because it makes me feel that I am full of energy	− 0.048	**0.638**	0.293	0.082	0.001	− 0.044
M11 …because it helps me to stay awake	− 0.020	**0.870**	− 0.069	− 0.049	− 0.066	− 0.027
M14 …because I become refreshed	0.021	**0.825**	0.132	− 0.040	0.031	− 0.002
M20 …because it makes me more motivated to work	− 0.032	**0.533**	*0.304*	0.159	− 0.036	− 0.030
M21 …because it peps me up	0.005	**0.494**	*0.376*	− 0.019	0.127	0.063
M25 …because it helps me to wake up	0.187	**0.689**	− 0.013	− 0.056	− 0.024	0.204
M27 …because it stimulates me	0.037	**0.824**	− 0.064	0.018	0.100	0.109
M30 …because sometimes it feels good to be stimulated	− 0.197	**0.502**	*0.303*	0.167	0.061	0.031
M34 …because it invigorates me	− 0.113	**0.774**	0.184	0.001	− 0.007	0.030
M37 …because I feel physically and mentally fitter	− 0.014	**0.670**	0.195	0.083	0.025	0.051
M6 ...because it improves my mood	0.083	0.033	**0.797**	− 0.087	− 0.002	0.047
M12 ...because my mood becomes better	0.092	0.144	**0.718**	0.007	0.031	0.013
M23 ... because it relieves tension	− 0.024	0.052	**0.493**	*0.300*	− 0.098	0.090
M18 …because I love the atmosphere associated with it	0.178	0.125	0.396	0.369	0.055	− 0.048
M32 …because it calms me down	0.160	− 0.013	0.394	0.267	− 0.044	0.031
M7 …because everyone in my company drinks it	− 0.024	− 0.105	0.134	**0.647**	0.024	− 0.013
M10 …because drinking coffee is important in social situations	0.087	− 0.008	0.092	**0.802**	− 0.216	− 0.010
M16 …because drinking coffee is a social event	0.151	0.033	− 0.029	**0.891**	− 0.157	− 0.007
M22 …because it is a pleasant addition to a good conversation	− 0.016	0.030	0.012	**0.783**	0.045	0.177
M31 …because it brings me together with other people	− 0.052	− 0.063	0.252	**0.731**	0.047	− 0.093
M36 …because it feels good with a conversation in company	− 0.004	− 0.009	− 0.056	**0.772**	0.093	0.221
M39 …it is good to relax with a cup of coffee	0.150	0.098	− 0.031	**0.552**	0.018	*0.269*
M3 …because I like its taste	0.103	− 0.018	0.098	− 0.046	**0.818**	− 0.053
M26 …because it is delicious	0.041	0.032	− 0.023	0.044	**0.905**	0.029
M35 …because I love the smell	0.158	0.030	− 0.038	*0.227*	**0.469**	*0.211*
M33 ...because it feels good to have a hot drink when it is cold	0.021	0.026	0.023	*0.239*	*0.314*	0.081
M5 …because it reduces headaches	0.029	− 0.093	0.098	− 0.002	− 0.073	**0.571**
M28 …because I have got used to it	*0.444*	0.023	0.043	− 0.028	0.039	**0.610**
M38 …because it is good for my blood pressure	− 0.104	0.129	0.105	0.086	− 0.007	**0.435**
M13 …because it feels good when I am smoking a cigarette	0.138	0.141	0.181	0.013	− 0.017	− 0.024
M17 …because it is good for my stomach	0.103	− 0.101	0.286	0.169	− 0.049	0.070
M19 …because it helps me sleep better	0.125	− 0.036	0.244	− 0.031	0.039	− 0.069
M29 …because it hydrates me	− 0.027	− 0.257	0.186	0.071	0.275	0.163

The highest factor scores which are above 0.40 are in bold. The second highest factor scores which indicate cross-loadings (factor loadings more than .32 on a second factor or the second highest loading has a difference less than 0.30 compared to the highest factor loading) are marked in italics

On the basis of the EFA on sample 1, the 6-factor solution with 23 items was tested on sample 2. As part of the CFA on sample 2, the modification indices were examined as well as the content of the items, and the error covariances in the CFA model were introduced. The items involved were similar in meaning and wording: item 2 (…because it helps when I am tired) with item 11 (…because it helps me to stay awake), item 10 (…because drinking coffee is important in social situations) with item 16 (…because drinking coffee is a social event) as well as item 10 with item 7 (…because everyone drinks in the company). After introducing error covariances, the CFA on sample 2 provided adequate fit indices (*χ*2 = 566.9, df = 212, *p* < 0.001; CFI = 0.912; TLI = 0.896; SRMR = 0.053; RMSEA = 0.074 [CI: 0.066–0.081]).

The previously established categories according to the results of the EFA and the CFA were modified. Habit, Symptom Management, Mood and Social were retained. The Habit factor includes items that characterize caffeine consumption as a ritual or a daily routine. Items of Symptom Management factor refer to the reduction of headaches and caffeine’s positive effect on blood pressure. The Mood factor includes items about optimizing mood with caffeine, and the Social factor includes items that imply the importance of caffeinated drinks in social settings. The Consumption factor (because of the taste or the smell of the beverage) was renamed to Taste, because only the items that related to the flavor of caffeinated beverages were retained. Other items—related to smell and temperature—were dropped. Ceasing Fatigue, Invigoration, and Improving Concentration appeared to belong to one factor that was named Alertness. The standardized loadings of the CFA are presented in Table 3.

**Table 3:** Confirmatory factor analysis of the MCCQ in sample 2 (*n* = 308)

	*Habit*	*Alertness*	*Mood*	*Social*	*Taste*	*Symptom Management*
M1 …because it is a ritual for me	0.663					
M8 …because it is an enjoyable habit	0.954					
M2 …because it helps when I am tired		0.789				
M4 …because it helps me to concentrate		0.816				
M9 …because it makes me feel that I am full of energy		0.84				
M11 …because it helps me to stay awake		0.808				
M14 …because I become refreshed		0.853				
M25 …because it helps me to wake up		0.759				
M27 …because it stimulates me		0.841				
M34 …because it invigorates me		0.816				
M37 …because I feel physically and mentally fitter		0.851				
M6 ...because it improves my mood			0.822			
M12 ...because my mood becomes better			0.915			
M7 …because everyone in my company drinks it				0.590		
M10 …because drinking coffee is important in social situations				0.762		
M16 …because drinking coffee is a social event				0.804		
M22 …because it is a pleasant addition to a good conversation				0.872		
M31 …because it brings me together with other people				0.763		
M36 …because it feels good with a conversation in company				0.852		
M3 …because I like its taste					0.889	
M26 …because it is delicious					0.954	
M5 …because it reduces headaches						0.659
M38 …because it is good for my blood pressure						0.870

### Correlations Between the Factors and Internal Consistency

Correlations between the factors (Table 4) ranged from 0.25 (between Taste and Symptom Management as well as Habit and Symptom Management) to 0.68 (between Habit and Taste). Further high correlations were found between Habit and Social and between Alertness and Mood, while a weak correlation was found between Alertness and Taste. Five factors had excellent Cronbach’s alphas and ranged between 0.81–0.95, while the Symptom Management factor—possibly due to the shortness of the scale—had a lower but acceptable Cronbach’s alpha (0.66).

**Table 4:** Correlations between the factors and internal consistency

	Habit	Alertness	Mood	Social	Taste	Symptom Management
Habit		.34	.47	.56	.68	.25
Alertness			.62	.35	.27	.47
Mood				.47	.36	.41
Social					.43	.40
Taste						.25
Cronbach’s alphas	.81	.95	.86	.91	.91	.66

*N* = 597. All correlations are significant at *p* < .001

### Caffeine Consumption Motives, Gender and Age

Comparing the means of the factors, Taste appeared to have the highest value (*M* = 3.47; *SD* = 1.32), followed by Habit (*M* = 2.67; *SD* = 1.35), Alertness (*M* = 2.60; *SD* = 1.13), Social (*M* = 1.93; *SD* = .95), Mood (*M* = 1.81; *SD* = 1.01) and Symptom Management (*M* = 1.67; *SD* = .98) motives. Differences in caffeine consumption motives between males and females and between two age groups are presented in Table 5. Women had higher scores on all factors and the difference was significant in the case of Habit, Social, Taste and Symptom Management. In relation to age, the younger group (18–24 years) had significantly higher scores in Alertness than the older group (25–68 years), and the older group had higher values in Habit and Symptom Management than the younger group (Table 5).

**Table 5:** Mean scores and standard deviations (in parentheses) of daily caffeine consumption and factors of the MCCQ related to gender and age (*N* = 598)

	Male (*n* = 171)	Female *(n* = 427)	*t* test (df)	Cohen d	18–24 years *n* = 363)	25–68 years (*n* = 226)	*t* test (df)	Cohen’s d
Daily caffeine use [mean (*SD*)]	180.16 (134.07)	223.52 (154.45)	3.21 (591)**	0.30	201.80 (156.92)	226.46 (137.75)	− 1.93 (582)	0.17
Habit	2.34 (1.24)	2.81 (1.36)	4.03 (340.22)***	0.36	2.58 (1.36)	2.84 (1.32)	2.27 (582)*	0.19
Alertness	2.50 (1.15)	2.64 (1.12)	1.30 (579)	0.12	2.74 (1.13)	2.36 (1.08)	4.00 (570)***	0.34
Mood	1.77 (1.01)	1.82 (1.01)	0.60 (591)	0.05	1.77 (.98)	1.86 (1.06)	0.97 (582)	0.09
Social	1.75 (0.87)	1.99 (0.97)	2.95 (342.18)**	0.26	1.89 (.95)	1.99 (.94)	1.30 (576)	0.11
Taste	3.30 (1.34)	3.54 (1.30)	1.98 (593)*	0.18	3.50 (1.36)	3.42 (1.25)	0.81 (503.55)	0.06
Symptom management	1.35 (0.70)	1.80 (1.04)	6.12 (453.45)***	0.51	1.52 (.85)	1.90 (1.11)	4.47 (382.47)***	0.38

*p < .05; **p < .01; ***p < .001

### Caffeine Consumption Motives and the Type of Caffeinated Beverage

In relation to the type of caffeinated products consumed, significant differences in every motive were detected (including dosage)—except for Taste (see in Table 6). The mixed group consumed significantly more caffeine than the other groups, while coffee as well as coffee and tea groups both consumed more caffeine than tea group and energy drink group. For Habit, the coffee group had significantly higher mean score, than the tea, energy drink, and mixed group, while the coffee and tea group had higher mean score than the tea group and energy drink group. For the Social motive, the tea group had lower mean score than the coffee and the coffee and tea group, while the energy drink group had lower mean score, than the coffee, the coffee and tea and the mixed group. For Mood and Alertness, the tea group had lower mean score than the coffee, coffee and tea and mixed groups. For Symptom Management, the energy drink group had lower mean score than the other four groups, and the tea group had lower mean score than coffee as well as coffee and tea group and the mixed group had lower mean score than the coffee group.

**Table 6:** Mean scores and standard deviations (in parentheses) of factors of the MCCQ related to the type of caffeinated product consumed (*N* = 598)

	Coffee (*N* = 215)	Tea (*N* = 152)	Energy drink (*N* = 19)	Coffee and tea (*N* = 87)	Mixed (*N* = 70)	*F* (df)
Daily caffeine intake (mg)	241.00^a^ (133.93)	179.52^b^ (132.16)	140.79^b^ (131.55)	239.53^a^ (110.45)	314.09^c^ (170.82)	12.07 (4)***
Habit	3.19 (1.27)^a^	2.41 (1.26)^b^	2.05 (1.18)^b^	2.98 (1.34)^ac^	2.46 (1.27)^bc^	11.30 (4)***
Alertness	2.76 (1.06)^a^	2.22 (1.08)^b^	2.73 (1.06)^ab^	2.90 (1.05)^a^	3.01 (1.14)^a^	9.21 (4)***
Mood	1.98 (1.06)^a^	1.51 (0.80)^b^	1.82 (1.25)^ab^	2.02 (1.09)^a^	1.94 (1.04)^a^	5.63 (4)***
Social	2.14 (0.96)^a^	1.71 (0.87)^bc^	1.42 (0.49)^b^	2.12 (0.92)^a^	1.91 (0.90)^ac^	7.22 (4)***
Taste	3.68 (1.16)	3.50 (1.35)	3.63 (1.49)	3.50 (1.28)	3.28 (1.21)	1.41 (4)
Symptom Management	1.96 (1.14)^a^	1.40 (0.73)^b^	1.08 (0.19)^c^	1.77 (0.89)^ad^	1.55 (0.76)^bd^	10.16 (4)***

Post-hoc analyses were performed with Games-Howell test. Means that have no superscript in common are significantly different from each other at *p* < .05

*p < .05; **p < .01; ***p < .001

## Discussion

The primary purpose of the present study was to develop a robust psychometric instrument for assessing caffeine consumption motives. A 6-factor solution emerged from a series of exploratory and confirmatory factor analyses, the results of which were mostly in line with the theoretically created categories. Three categories—ceasing fatigue, invigoration and improving concentration—were highly overlapping and finally merged into one factor (Alertness) confirming the results of Graham (1988). The category of consumption (taste or the smell of the beverage) also narrowed down to one factor (Taste), probably because different caffeinated products are distinctive in both these respects. While all caffeinated beverages have a distinctive flavor, their scents are not always distinctive (as in the case of energy drinks). Moreover, the consumption of caffeine pills was so rare in the sample (only 0.6% of the participants consumed caffeine this way) that it had no real influence over this factor.

Although several factors—Alertness, Mood, Social and Taste—have been noted in previous studies (Graham 1988), Habit and Symptom Management were new factors that emerged in the present study. By examining the items of Symptom Management more closely, it was evident that they referred to specific states such as caffeine’s perceived effects on blood pressure or headaches. Interestingly, these perceived effects can be reduced as well as triggered by caffeine (Shapiro 2007). Therefore, this factor can be associated with a specific pattern of caffeine use and caffeine-related problems when compared to other motives. Experiencing headaches can also be a consequence of caffeine withdrawal (American Psychiatric Association 2013); therefore, this factor is associated with physiological symptoms of substance use disorder. Interestingly, Habit and Symptom Management had a relatively low correlation with each other although both factors are implicated in the presence of caffeine dependence symptoms. This finding indicates that caffeine use disorder and the various underlying motives should undergo further empirical examination. Alertness also appeared to be an important motive. Caffeine consumption can reduce fatigue and improve performance on vigilance tasks especially when alertness is reduced (Smith 2002). However, mental alertness and mental performance are not improved when caffeine consumption becomes frequent (Rogers et al. 2013). Consuming caffeine to improve alertness can be especially desirable for those who need to be awake (e.g., shift workers). Although caffeine combined with short naps can improve cognitive performance, shift workers may be at greater risk of sleep disturbance due to prior caffeine use (Wright et al. 2013), and those who are dependent upon caffeine show poorer sleep quality (Ogeil and Phillips 2015).

It should also be noted that since the construction of the Motives for Caffeine Consumption Questionnaire, another instrument—the Caffeine Motives Questionnaire (CMQ) (Irons et al. 2014)—has also been developed to measure the motives of caffeine consumption. Though both questionnaires have acceptable fit indices, they have different factor structures: the CMQ has a 4-factor structure (Irons et al. 2014) while the MCCQ has a 6-factor structure. Another difference is that while the CMQ was based purely on theoretical consideration, the MCCQ additionally applied an inductive method. It would therefore be beneficial for future studies to compare the two instruments.

The analyses demonstrated the different role of the underlying motives for gender, age, and the type of the caffeinated products. Some differences between men and women were found. In general, female participants had higher scores on each factor, and the differences were significant for Habit, Social and Taste with lower effect sizes, and for Symptom Management with medium effect size. This indicates that women have higher motivation in general to use caffeine compared to men, although the underlying reasons are unclear. One possible explanation for the higher score on Social factor is that women tend to have more communal traits than men (Abele 2003). However, it is important to mention that the sample was predominantly female (71.5%) which could result in some biases in relation to the findings obtained. Furthermore, female participants had higher daily caffeine consumption which may also have led to higher scores on some motives for caffeine consumption. Therefore, confirmation of gender differences requires further examination in future studies.

The younger age group had higher mean scores on Alertness, while the older age group had higher mean scores on Habit and Symptom Management. This could point to the fact that changes from positive to negative reinforcement may occur as individuals move from an impulsive behavior (like consuming caffeine for its stimulant effect) to compulsive behaviors (such as more automatic consumption of caffeine and consumption in order to avoid withdrawal) (Koob 2004) that may happen when an individual has a longer history with caffeine. However, it should be noted that the older adult group had a wider age range in order to have a robust sample size for psychometric analyses. Consequently, the role of negative reinforcement may be even more important at the older end of this age group.

The MCCQ is applicable for use among the different kinds of caffeine consumers. Five of six motives for caffeine consumption differed among the five types of caffeine consumers. However, Taste appeared to be equally and highly important for all types of caffeine consumers. This can be considered an important finding with regard to the popularity and maintenance of caffeine consumption. Previous studies have shown that the liking of a new drink increased after a few repeated exposures to the drink if it contained caffeine and the participants were acutely caffeine withdrawn (Rogers et al. 1995; Yeomans et al. 1998). It is proposed that this learned flavor preference develops as a result of association of the flavor of the drink with the negative reinforcing effects of caffeine (Rogers et al. 1995). The results of the present study indicate that learned flavor preference reinforced by caffeine is equally important for the different types of caffeinated beverages (e.g., coffee, tea and energy drinks) despite their differing tastes.

The coffee group as well as the coffee and tea group had relatively higher means on the Habit factor that might indicate a higher degree of caffeine dependence in coffee consumers compared to the consumers of other kinds of caffeinated products. Another notable finding was the relatively low agreement with Habit as a motive for the Energy drink group. This is consistent with the use of energy drinks for special occasions such as partying or completing a major educational project (Malinauskas et al. 2007) and suggests that energy drink users may have an irregular, in other words less habitual, consumption pattern compared to other groups of caffeine consumers.

The same pattern of scores emerged for Symptom Management. The item which referred to headache (*M* = 1.6, SD = 1.06) and the item which referred to blood pressure (*M* = 1.74, SD = 1.2) had quite similar ratings which suggests that both of them are important when considering the bodily sensations associated with caffeine consumption. Headache is a characteristic symptom of caffeine withdrawal (Juliano and Griffiths 2004) and lower blood pressure can also be the consequence of acute caffeine withdrawal (James 1994). To date, the evidence for the existence of the latter effect is not convincing (Juliano and Griffiths 2004); however, it can be assumed that the Symptom Management factor related mainly to the physical aspects of caffeine consumption and caffeine withdrawal, especially for coffee consumers.

The tea and the energy drink consumers scored lower on the Social factor which underpins coffee’s importance in social settings. For energy drink consumers who had very low scores on this factor, a possible explanation is that they use energy drinks in particular settings—such as for insufficient sleep, energy enhancement in general, studying for an exam or to complete a major project, driving a car for a long time, drinking it with alcohol while partying or to treat a hangover (Malinauskas et al. 2007)—which are generally unrelated to social situations. Tea consumers had lower scores on the Alertness and Mood motives which is consistent with the lower concentration of caffeine in tea compared to coffee and energy drinks (e.g., Chin et al. 2008). Therefore, tea consumers are less likely to detect the stimulating and altering effects of caffeine compared to the other groups.

An interesting finding was the similarity of the coffee group and the coffee and tea group in all motives. This indicates that the consumption of coffee in the coffee and tea group has a greater impact on motives than the consumption of tea. However, these results may also be related to the dosage of caffeine. For example, the lower scores on several motives for tea and energy drink users may be explained by the lower caffeine dosages consumed in these particular products compared to coffee drinkers. The similarities and differences between the various groups of caffeine consumers make it necessary to examine the pattern of caffeine consumption and the possible latent groups of caffeine consumers more thoroughly taking into account both the type of caffeinated beverage and the frequency of caffeine use.

### Limitations and Future Research Directions

The models explored in the present paper had acceptable fit indices and the factors were theoretically and empirically interpretable based on previous literature. The two samples in the study were similar regarding the basic demographic characteristics apart from age and average daily caffeine consumption. Although the lower average consumption of sample 1 may have led to biases in the findings, it is important to mention that both samples and also the sample in the pilot study had a higher daily caffeine use compared to participants in a study using a representative sample in Hungary (Szeitz-Szabó et al. 2011). Therefore, it is necessary to evaluate the MCCQ in larger samples that include individuals with lower average caffeine consumption, and individuals who consume caffeine only occasionally, as well as teenagers because the initiation of caffeine use mostly occurs during adolescence (Kendler et al. 2008). Consumers of energy drinks, cola drinks, and caffeine pills were possibly underrepresented in the present study, therefore, it is important to include these caffeine consumers in future studies. Prevalence of energy drink use among adolescents is around 6% in Hungary (Visram and Hashem 2016) which may exceed the prevalence observed among adults. Consequently, the examination of the adolescent population could be especially advantageous.

The estimation of caffeine content was based on general estimations but future studies should be more specific in estimating the caffeine content of specific brands (e.g., McCusker et al. 2003; Mitchell et al. 2014) with respect to products available in Hungary and elsewhere. This would reflect somewhat on the characteristics of caffeine consumption in Central and Eastern Europe. The MCCQ also needs to be validated in different cultural settings because the predominant sources of caffeine (and therefore caffeine consumption motives) can vary in different regions and countries (e.g., Fulgoni et al. 2015; Gibson and Shirreffs 2013; Mineharu et al. 2009; Mitchell et al. 2014; Radhika et al. 2011). The results presented here do not demonstrate causal relationships because of the cross-sectional nature of the data collected. The convenience sampling also reduces the generalizability of the findings. An important objective of future research is to further explore the possible latent groups of caffeine consumers—on the basis of type of beverage and/or the quantity of daily use—to accurately detect the possible differences in the pattern of consumption, problematic use and underlying motives.

The MCCQ appears to be a robust tool with good overall psychometric properties for assessing caffeine consumption motives. As motives can be important predictors of substance use (e.g., Kuntsche et al. 2007; Stewart and Zack 2008; Vajer et al. 2011), the analysis of the relationship between the various caffeine consumption motives (assessed by the MCCQ) and the symptoms of caffeine withdrawal and caffeine use disorder could be a focus of future empirical investigations. Further cross-sectional as well as clinical studies would confirm the reliability and validity of the questionnaire. Additionally, longitudinal studies could be employed to systematically examine causal determinants of caffeine consumption over time and the extent to which motives for caffeine consumption change over time.

## Appendix

**Table 7:** Motives for Caffeine Consumption Questionnaire. People drink caffeinated beverages for different reasons. The list below contains several reasons. Please, indicate for each item how often do you drink caffeinated beverages due to that specific reason. There are no right or wrong answers. We are just interested in why you drink these beverages. When answering the questions, please take into account every caffeinated product you consume! This includes coffee, tea, energy drinks, caffeine pills, etc.

I drink caffeinated beverages…	Never / almost never	Sometimes	Half of the cases	Most of the time	Almost every time /always
1. …because it is a ritual for me	1	2	3	4	5
2. …because it helps when I am tired	1	2	3	4	5
3. …because I like its taste	1	2	3	4	5
4. …because it helps me to concentrate	1	2	3	4	5
5. …because it reduces headaches	1	2	3	4	5
6. ...because it improves my mood	1	2	3	4	5
7. …because everyone in my company drinks it	1	2	3	4	5
8. …because it is an enjoyable habit	1	2	3	4	5
9. …because it makes me feel that I am full of energy	1	2	3	4	5
10 …because drinking coffee (or other caffeinated beverages) is important in social situations	1	2	3	4	5
11. …because it helps me stay awake	1	2	3	4	5
12. ...because my mood becomes better	1	2	3	4	5
13. …because I become refreshed	1	2	3	4	5
14. …because drinking coffee (or other caffeinated beverages) is a social event	1	2	3	4	5
15. …because it is a pleasant addition to a good conversation	1	2	3	4	5
16. …because it helps me to wake up	1	2	3	4	5
17. …because it is delicious	1	2	3	4	5
18. …because it stimulates me	1	2	3	4	5
19. …because it brings me together with other people	1	2	3	4	5
20. …because it invigorates me	1	2	3	4	5
21. …because it feels good with a conversation in company	1	2	3	4	5
22. …because I feel physically and mentally fitter	1	2	3	4	5
23. …because it is good for my blood pressure	1	2	3	4	5

**Factors**:

Habit: 1, 8

Alertness: 2, 4, 9, 11, 13, 16, 18, 20, 22

Mood: 6, 12

Social: 7, 10, 14, 15, 19, 21

Taste: 3, 17

Symptom Management: 5, 23
